# Rapid Biomarker-Based Diagnosis of Fibromyalgia Syndrome and Related Rheumatologic Disorders by Portable FT-IR Spectroscopic Techniques

**DOI:** 10.3390/biomedicines11030712

**Published:** 2023-02-27

**Authors:** Siyu Yao, Haona Bao, Shreya Madhav Nuguri, Lianbo Yu, Zhanna Mikulik, Michelle M. Osuna-Diaz, Katherine R. Sebastian, Kevin V. Hackshaw, Luis Rodriguez-Saona

**Affiliations:** 1 Department of Food Science and Technology, Ohio State University, Columbus, OH 43210, USA; 2 Center of Biostatistics and Bioinformatics, Ohio State University, Columbus, OH 43210, USA; 3 Department of Internal Medicine, Division of Rheumatology, The Ohio State University, 480 Medical Center Drive, Columbus, OH 43210, USA; 4 Department of Internal Medicine, Dell Medical School, The University of Texas, 1601 Trinity St., Austin, TX 78712, USA; 5 Department of Internal Medicine, Division of Rheumatology, Dell Medical School, The University of Texas, 1601 Trinity St., Austin, TX 78712, USA

**Keywords:** fibromyalgia, biomarker, disease diagnostics, portable FT-IR spectroscopy, chemometrics, blood

## Abstract

Fibromyalgia syndrome (FM), one of the most common illnesses that cause chronic widespread pain, continues to present significant diagnostic challenges. The objective of this study was to develop a rapid vibrational biomarker-based method for diagnosing fibromyalgia syndrome and related rheumatologic disorders (systemic lupus erythematosus (SLE), osteoarthritis (OA) and rheumatoid arthritis (RA)) through portable FT-IR techniques. Bloodspot samples were collected from patients diagnosed with FM (n = 122) and related rheumatologic disorders (n = 70), including SLE (n = 17), RA (n = 43), and OA (n = 10), and stored in conventional protein saver bloodspot cards. The blood samples were prepared by four different methods (blood aliquots, protein-precipitated extraction, and non-washed and water-washed semi-permeable membrane filtration extractions), and spectral data were collected with a portable FT-IR spectrometer. Pattern recognition analysis, OPLS-DA, was able to identify the signature profile and classify the spectra into corresponding classes (Rcv > 0.93) with excellent sensitivity and specificity. Peptide backbones and aromatic amino acids were predominant for the differentiation and might serve as candidate biomarkers for syndromes such as FM. This research evaluated the feasibility of portable FT-IR combined with chemometrics as an accurate and high-throughput tool for distinct spectral signatures of biomarkers related to the human syndrome (FM), which could allow for real-time and in-clinic diagnostics of FM.

## 1. Introduction

Fibromyalgia (FM) is a common chronic disease characterized by widespread pain, cognitive problems, sleep disturbances, and chronic fatigue, along with a plethora of other symptoms [1]. This disease affects between 2 and 5% of the population or approximately 15 million people at any one time in the United States alone [2]. The coalescence of psychiatric comorbidity coupled with physical and/or functional symptomatology frequently results in the exacerbation of underlying depressive symptoms, which makes treatment more challenging [3]. FM represents a significant burden on health resources, leading to voluminous expenditures in health, social, and economic sectors. In addition, work absenteeism or disability may lead to job loss and the use of government assistance, further burdening society due to the care of affected individuals [4].

The burden that FM causes on patients, relatives, and society can be reduced by early diagnosis and treatment [5]. FM diagnosis has evolved over the years, changing from dependence on the presence of tender points to a method that is inclusive of comorbid features. Current diagnostic criteria, which rely on a combination of scores from the widespread pain index (WPI) and a symptom severity (SS) scale, help take into consideration the totality of how FM affects the individual [6,7]. Nevertheless, rheumatologic disorders, including rheumatoid arthritis (RA), systemic lupus erythematosus (SLE), and osteoarthritis (OA), may co-exist with FM or have overlapping symptoms and psychosocial features, which can confound the diagnosis and treatment of both conditions [8,9,10]. Furthermore, patients with poorly explained symptoms are often lumped into the FM category inappropriately by physicians that lack training or experience [11].

As the etiopathogenesis of FM is still poorly understood, there are currently no biomarkers or reliable, objective diagnostic tests for FM [7,12]. Up to 75% of patients go undetected with FM, resulting in postponed care due to the absence of distinct diagnostic markers [7,12]. In addition, FM patients make up a substantial proportion of chronic pain patients receiving opioids, even though medical guidelines recommend against their use in FM [13,14]. Unnecessary exposure of FM patients to opioids may result in poorer outcomes and addiction and makes patients prone to numerous adverse events, including death [14,15].

Developing a reliable and objective earlier diagnostic method would be a significant step towards improving the health conditions of patients, lowering healthcare costs, and improving the quality of life for individuals with FM. Infrared fingerprinting capabilities allow for fast and high-throughput analysis of a wide range of sample types and provide distinctive chemical ‘fingerprints’ with a unique spectral profile [16]. Mass spectrometry (MS) techniques and NMR spectroscopy could provide enough selectivity and specificity for screening metabolites [17]. However, high-cost instrumentation, labor-intensive and complicated sample preparation, and well-trained personnel are required to operate the instrumentation, which makes them less amenable to be implemented in clinics [18]. In the last decades, portable infrared (IR) spectrometers have become commercial, with developments in micro-electro-mechanical system (MEMS) production and optoelectronics [19]. Portable optical systems have incorporated the analytical precision of spectroscopy into in situ/in-field applications with high spectral resolution equivalent to benchtop instruments for chemical identification [18]. The application of chemometrics, such as orthogonal partial least squares discriminant analysis models (OPLS-DA), soft independent modeling class analogy (SIMCA), and support-vector machines (SVM), is critical to extract unique spectral features from other predominant vibrations related to chemical and physical properties of biological samples [20]. Recent studies have investigated FT-IR techniques combined with chemometrics for disease diagnostics [18], such as psoriasis [21], cancer [22,23], and Alzheimer’s disease [24]. 

Our group reported the first metabolomics approach in diagnosing FM and related rheumatologic disorders (RA, OA and SLE) [5]. The low-molecular-weight fraction (LMF) of human blood was isolated (filtrate) by using centrifugal ultrafiltration on a semi-permeable membrane (30 K), while high-molecular-weight solutes were retained (retentate) [5]. Pattern recognition analysis of the spectra allowed discriminating FM patients from RA and OA groups that appeared to be metabolically similar. However, chemical characterization of the serum fraction by using a Raman database revealed that the spectrum was dominated by glycerol bands. Raman analysis of the Whatman bloodspot blank paper and the ultrafiltration membranes indicated that the samples contained glycerol that was carried over from the ultrafiltration membranes. Glycerol is coated to the membranes to maintain open pores and preserve the membrane before use [25]; however, it dissolved during the extraction process and accumulated in the serum fraction. This artifact masked several portions of the IR spectra except for the amide region (1700–1500 cm^–1^) that served to discriminate the subjects based on their disease. Nonetheless, alternative sample preparation methods are required to avoid the artifact. Pure blood samples contain large molecules such as proteins, which may obscure some useful information [26]. Protein precipitation is usually achieved by using an organic solvent such as acetonitrile (ACN) with great reproducibility and high efficiency compared with using acidic reagents or heat [27]. Physical extraction is another common approach and includes semi-permeable filtration methods [28]. 

Few studies have been carried out to find biomarkers for FM diagnosis recently. Hackshaw et al. evaluated vibrational spectroscopy on distinguishing FM (n = 50) patients from patients with other rheumatological diseases (RA (n = 29), OA (n = 19), and SLE (n = 23)) and reported that protein backbones and pyridine-carboxylic acids could be key metabolites for disease discrimination [5]. Some other studies compared metabolomic differences between FM patients and healthy controls. Caboni et al. assessed distinct metabolomic profiles of blood plasma from FM patients (n = 22) and controls (n = 21) using LC-Q-TOF/MS analyses, which revealed that FM patients have a greater level of lysophosphocholines than controls [29]. Malatji et al. investigated the metabolite profile of urine from FM patients and healthy controls using NMR and suggested FM patients (n = 18) could have increased metabolites that are widely related to the gut microbiome [30]. However, no reproducible, dependable distinguishing biomarkers for any of the techniques have been found previously [12]. Furthermore, none of these studies have employed a large sample number, including more variance from subjects, which is generally required in disease diagnosis studies [18,26].

The objective of this study was to develop a non-targeted fingerprinting FT-IR technique that can identify FM as distinct from other related rheumatologic disorders. Ultimately, the development of robust predictive models based on portable IR spectra could allow for real-time and in-clinic diagnostics for potential revolutionary advances and huge cost savings.

## 2. Materials and Methods

### 2.1. Patient Sample Recruitment and Sample Storage

All studies involving human subjects were approved by The University of Texas at Austin institutional review board and abided by the Declaration of Helsinki principles. Following IRB approval (study no. 2020030008/approval date 19 June 2020), blood samples were obtained from patients with FM (n = 122) and related rheumatologic disorders (n = 70), including SLE (n = 17), RA (n = 43), and OA (n = 10), at the University of Texas at Austin and the Ohio State University rheumatology clinics located at Care Point East in Columbus, Ohio. Bloodspots were obtained at the University of Texas at Austin Rheumatology Clinics and at The Ohio State University Care Point East Rheumatology clinics from September 2020 to January 2023.

Patients’ blood samples were collected and stored on bloodspot cards (Whatman 903 Protein Saver Snap Apart Card, GE Healthcare, Westborough, MA, USA) at −20 °C until they were shipped to the Rodriguez-Saona Vibrational Spectroscopy Laboratory at The Ohio State University Department of Food Sciences on dry ice and stored for analysis. The bloodspot size was standardized by collecting samples on cards with preprinted circles as guides, with each circle containing approximately 50 μL of blood. 

Self-reported symptoms were obtained from all subjects using the Revised Fibromyalgia Impact Questionnaire (FIQR), a 10-item self-rating instrument that measures physical functioning, work status, depression, anxiety, sleep, pain, stiffness, fatigue, and well-being [31]. The Beck Depression Inventory (BDI) is a 21-item questionnaire used to quantify the psychological/behavioral dimension of depression [32]. The Symptom Impact Questionnaire Revised (SIQR) is the FM-neutral version of the FIQR and does not assume the patient has FM [33].

Criteria for the diagnosis of FM included: age 18–80 with a history of FM and meeting current American College of Rheumatology (ACR) criteria [6]. The diagnosis of OA [34], RA [35], and SLE [36] was based on ACR criteria for each disorder. 

Sigmaplot v14.5 and SigmaStat v4.0 software,(Inpixon, Palo Alto, CA, USA) were utilized for statistical analysis of questionnaires and calculation of correlation coefficients. 

### 2.2. Sample Preparation

Samples were prepared by four approaches for spectral acquisition. (a) Blood serum aliquots: one circle was punched from the bloodspot card, diluted with 1 mL of water (HPLC grade, Sigma-Aldrich, Inc., St. Louis, MO, USA) in a 15 mL centrifuge tube, and mixed by sonication (Sonic Dismembrator Model 100, Fisher Scientific, Inc., Pittsburgh, PA, USA) for 15 min. Then, 100 μL of the diluted blood fluid was dried as a film using a vacuum centrifuge (Vacufuge plus Concentrator, Eppendorf, Inc., Westbury, NY, USA) and used for further analysis. (b) Chemical protein precipitation extraction [37]: one circle of the bloodspot was mixed with 1 mL water with the same approach described above in a 15 mL centrifuge tube. Then, 1 mL of blood fluid was mixed with 4 mL acetonitrile (HPLC grade, Sigma-Aldrich, Inc., St. Louis, MO, USA), and the mixture was vortexed thoroughly. After that, the centrifuge tube stayed in the fridge at 4 °C for 1 h and centrifuged (Sorvall Legend XFR Centrifuge, Thermo Fisher Scientific, Inc., Waltham, MA, USA) at 4000 rpm for 15 min at 4 °C to precipitate proteins. The supernatant was collected and dried as a film by using a sample concentrator (BTLab 103 Systems, BenchTop Lab System, St. Louis, MO, USA) with nitrogen. To remove most of the protein (i.e., hemoglobin) thoroughly, the dried film was redissolved by 100 μL water (HPLC grade) and mixed with 400 μL acetonitrile (HPLC grade, Sigma-Aldrich, Inc., St. Louis, MO, USA) to participate with the remaining protein and centrifuged to obtain the supernatant part. Finally, the supernatant part was evaporated into a film by a sample concentrator.

Another two extraction approaches were conducted by using semi-permeable membrane ultrafiltration extraction procedures by Hackshaw et al. with minor modifications [5]. (c) Washed semi-permeable membrane filtration extraction: Amicon Ultra-4 (10 K) centrifugal filter tubes (Sigma-Aldrich, Inc., St. Louis, MO, USA) were washed 4 times (3 mL, each time) with water (HPLC grade) by centrifuging at 4000 rpm for 15 min at 4 °C to eliminate the glycerol coated on the walls of the filter. One circle of the bloodspot was mixed with 1 mL water with the same approach described in (a). Then, the supernatant was transferred to the washed Amicon filter tube and centrifuged at 4000 rpm for 15 min at 4 °C. Blood filtrate fluid was concentrated into a film by a sample concentrator. The low-molecular-weight fraction (LMF) of the human plasma proteome, obtained by centrifugal membrane filter devices, is a significant source in identifying plasma-based biomarkers of disease [28]. Overall, semi-permeable membrane filters removed proteins and isolated LMF of water-soluble molecules (i.e., amino acids, peptides, sugars and lipids). (d) Semi-permeable membrane filtration extraction: in this approach, filters did not wash before adding the dissolved blood and all the rest of the procedures followed the same as described above in (c). Therefore, the samples extracted by this approach contained the artifact glycerol. 

### 2.3. Spectral Data Acquisition

**Figure 1:**
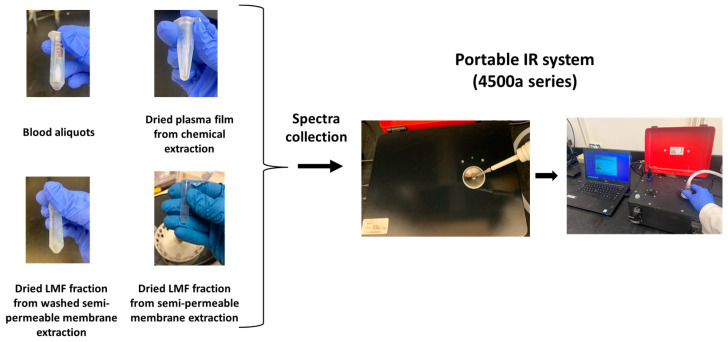
Spectral acquisition of prepared blood samples using a portable FT-IR sensor coupled with triple-reflection diamond attenuated total reflectance (ATR) crystal.

A 4500a series Agilent’s portable FT-IR unit (Agilent Technologies, Inc., Santa Clara, CA, USA) equipped with 3 bounce diamond attenuated total reflectance (ATR) was utilized for spectral acquisition, covering the spectral range from 4000 to 700 cm^−1^. It has a 200 µm active area on a 2 mm diameter sampling surface, giving ~6 µm penetration depth, and is equipped with a zinc selenide beam splitter, a high-throughput Michelson interferometer, and a thermoelectrically-cooled dTGS detector [38]. Dried blood fluid aliquots and plasma pellets (extracted by using acetonitrile and washed semi-permeable membrane) were redissolved in 10 µL of HPLC grade water and vortexed for 15 s to mix thoroughly for spectral acquisition, while samples with glycerol extracted by using the non-washed membrane method did not have to be redissolved. Then, 2 µL of the prepared sample was applied directly onto the ATR sampling window, and the excess water was dried under the vacuum to obtain a dry, thin film on the sampling window (Figure 1). The sampling window was cleaned with 70% ethanol, and a background was obtained after every reading. To enhance the signal-to-noise ratio, 128 scans were co-added with 8 cm^−1^ resolution for spectral collection. Spectra were recollected, or samples were re-extracted from blood spots when spectral inconsistencies were encountered. Collected spectra were recorded using the Agilent MicroLab PC software (Agilent Technologies, Inc., Danbury, CT, USA).

### 2.4. Multivariate Data Analysis

IR spectral differences between samples from subjects with FM and related rheumatologic disorders (SLE, RA, and OA) were analyzed using multivariate data analysis to resolve the information of interest and cluster the samples according to the assigned sample class (health condition) [5]. The spectral data were randomly divided into a training (75%, FM (n = 92) and non-FM (RA, OA and SLE) (n = 53)) and an independent external validation (25%, FM (n = 30) and non-FM (RA, OA and SLE) (n = 17)) set to generate the predictive algorithm for diagnosing FM and related rheumatologic disorders. The spectra were imported into the Pirouette pattern recognition software (Pirouette version 4.5, Infometrix Inc., Woodville, WA, USA) from the portable 4500a FTIR instrument as GRAMS (.spc) files to perform orthogonal signal correction-partial least squares discriminant analysis (OPLS-DA) analysis. Spectral data were transformed by the Savitsky–Golay (SG) second derivative (21 points for spectra of blood aliquots, protein-precipitated samples, and LMF samples, and 7 points for spectra of LMF samples with glycerol) and further preprocessed by mean centering. SG filtering enhanced minor bands, resolved overlapping bands, and suppressed unwanted spectral features (i.e., scattering effects), and mean centering helped to alleviate multicollinearity [39]. 

OPLS-DA is a supervised learning technique that relates IR fingerprinting data to the known information of class membership, such as FM (class 1) and related rheumatologic disorders (SLE, RA, and OA, class 2), to build up the training models, elucidating separation between the groups [40]. Orthogonal signal correction (OSC), a data filtering technique, was used to remove systematic spectral variation that did not agree with the assigned group memberships and to minimize the variance between individuals [40,41]. The PLS-DA technique extracted factors from both X and Y such that the covariance between the extracted factors was maximized. The discriminating ability of each OPLS-DA model was evaluated using two validation approaches. Firstly, the internal cross-validation of each OPLS-DA model’s performance was assessed using a leave-one-out approach, whereby each sample, in turn, was excluded, and a model was generated from the remaining samples to predict the class membership of the excluded sample. This internal cross-validation approach can provide the performance of the training model with the diagnostics statistics (misclassification and R). R represents the “goodness of fit” [42]. The optimal number of latent variables (LVs) were selected by applying the cross-validation approach, while the results of cross-validated OPLS-DA represented the classification of samples in the training set. Secondly, the external validation of each training model was assessed by the independent external validation set (25%), unseen by the training model, which provided an unbiased predictive accuracy, sensitivity and specificity performance, resembling in-clinic applications.

## 3. Results

### 3.1. Clinical Characteristics of Subjects

Widespread pain syndromes such as FM, RA, SLE and OA are common problems in the general population, but the pathogenesis of these disorders varies greatly and, particularly for FM, is not well understood. In addition, these disorders may frequently overlap, making diagnosis even more challenging. Therefore, a sensitive and specific diagnostic test for FM would be a significant advance and is currently an unmet need. The clinical characteristics of the patients are presented in Table 1.

Table 1 shows that patients with FM (n = 122, F: 114, M: 8) had a mean age of 44.5 +/−13.2 with a range of 18–73. Their BMI was 32.3+/−9.4, with a mean FIQR of 54.9+/−18.2 and a mean BDI of 19.5+/−9.3. Patients with RA (n = 43, F: 33, M: 10) had a mean age of 54.6+/−13.4 with a range of 20–77. Their BMI was 31.4+/−8.2, with a mean SIQR of 34.6+/−25.4 and a mean BDI of 9.5+/−7.9. Patients with SLE (n = 17, F: 16, M: 1) had a mean age of 43.9+/−15 with a range of 18–68. Their BMI was 29.9+/−8.9, with a mean SIQR of 35.4+/−28.9 and a mean BDI of 10.9+/−10.7. Finally, patients with OA (n = 10, F: 7, M: 3) had a mean age of 63.5+/−8.0 (range 52–79), BMI of 35.8+/−9.9, with a mean SIQR of 27.4+/−18.9 and a mean BDI of 7.3+/−6.3.

**Figure 2:**
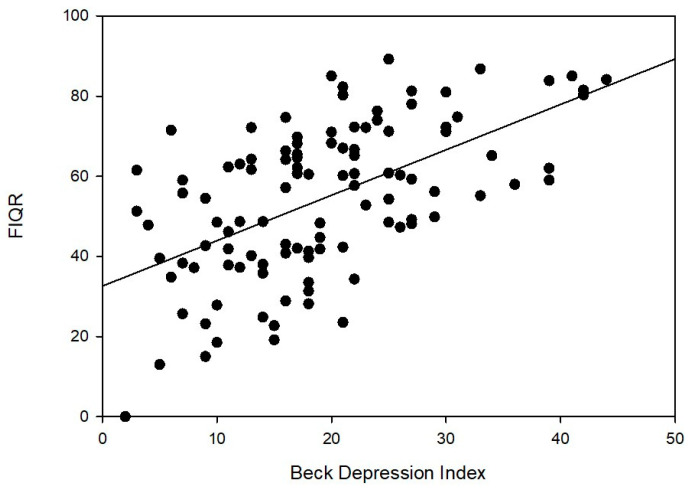
FIQR versus BDI for FM subjects. Pearson correlation is 0.588. Statistically significant at the 0.01 level (two-tailed).

Scatterplot analyses of the Fibromyalgia Impact Questionnaire Revised (FIQR) vs. the Beck Depression Index (BDI) for subjects with FM are presented in Figure 2. Corresponding Pearson coefficients and *p*-values are +0.588 with *p* < 0.01.

### 3.2. IR Spectroscopy

**Figure 3:**
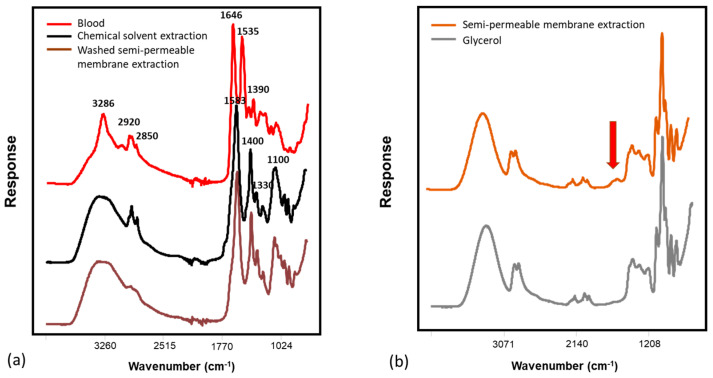
FT-IR representative spectra of (**a**) blood aliquot, plasma extracted by a chemical solvent precipitation method and low-molecular-weight fraction (LMF) of human blood obtained by a washed semi-permeable membrane extraction method, and (**b**) glycerol and plasma extracted by a non-washed semi-permeable membrane extraction approach, collected by a portable 3-reflection ATR system (4500a series); The red arrow marked the main spectral difference.

Figure 3a shows the spectra collected from blood aliquots, plasma extracted by a chemical protein-precipitated method, and the low-molecular-weight fraction (LMF) of human blood extracted by a water-washed semi-permeable membrane filtration method. The broad peak centered around 3200–3300 cm^−1^ was primarily from -OH stretches, which could also be associated with -NH stretches. The peaks occurring in the region 2970–2840 cm^−1^ were attributed to methylene and methyl -CH stretching that are present in hydrocarbon chains of lipids, proteins, and other metabolites [43]. Spectral features of chemically extracted plasma and washed-membrane-extracted LMF showed similar profiles. A strong peak was observed at 1583 cm^−1^ with a slight shoulder at 1670 cm^−1^. These were associated with amide II (N-H in-plane bend and C-N stretch) [44] and amide I bands (C = O stretch), which were related to the peptide backbone conformation [45]. The peaks between 1000 and 1200 cm^−1^ were attributed to C-O single bonds in carbohydrates [46]. Overall, both the chemically precipitated and the LMF extraction methods mainly removed large proteins and isolated hydrophilic molecules (i.e., amino acids, peptides, sugars, and lipids), therefore, resulting in a remarkable decrease in some IR absorption bands in the region between 1400 and 1800 cm^−1^ compared with the spectral profile of the blood aliquots [47]. For example, the absorbances of bands centered at 1646 and 1535 cm^−1^, which were associated with the amide I and II characteristics of large proteins (i.e., hemoglobin), were more evident in the spectra of blood aliquots [48,49]. In Figure 3b, spectral features of samples extracted by a non-washed semi-permeable membrane were dominated by glycerol bands, except the amide region (1700–1500 cm^−1^). Glycerol is coated on the membranes to maintain open pores and to preserve the membrane before use [50].

### 3.3. OPLS-DA Prediction Model Development for Diagnosis of Fibromyalgia and Other Rheumatologic Disorders

**Figure 4:**
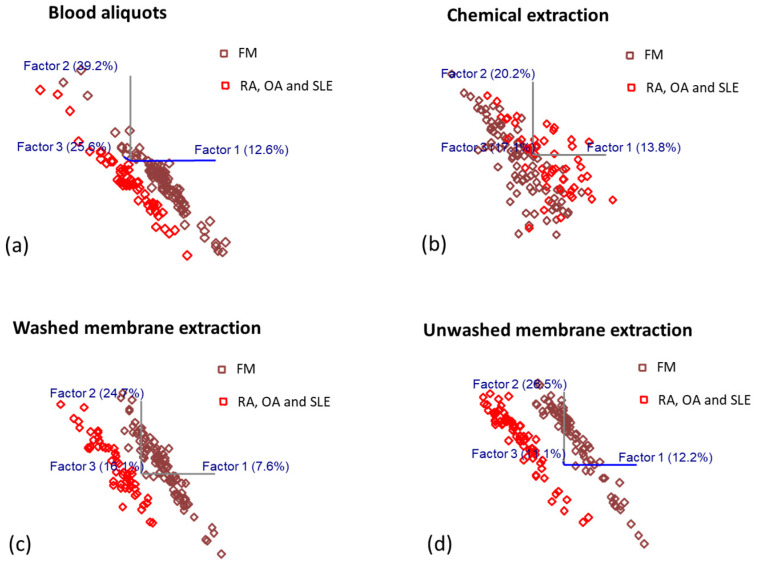
The score plots with the first three latent variables (LVs) of OPLS-DA regression models obtained from the spectral data of samples prepared by (**a**) redissolving blood aliquots, (**b**) chemical extraction, (**c**) washed membrane extraction (LMF), and (**d**) unwashed membrane extraction (LMF with glycerol).

As described above, OPLS-DA analysis was performed to generate predictive algorithms for the diagnosis of fibromyalgia and other rheumatologic disorders by combining IR spectral data with the known information of class membership. To evaluate the robustness of the predictive models, 75% of the spectral data were randomly selected and allocated as the calibration set, and the remaining 25% of data was assigned as the external validation set. The optimum latent variable (LV) numbers of predictive algorithms were determined by leave-one-out cross-validation in order to discriminate between the two classes and minimize the overfitting issue [39]. Highly collinear neighboring wavenumbers were excluded from the whole spectrum to maximize the predictive performance of the calibration models. The score plots corresponding to the first three latent variables (LVs) of OPLS-DA regression models obtained from the spectral data of samples prepared by (a) redissolving, (b) chemical precipitation extraction, (c) washed membrane extraction and (d) unwashed membrane extraction were presented in Figure 4. The score plots showed distinctive clusters of spectra from subjects with fibromyalgia and subjects with other rheumatologic disorders. To generate the cross-validated calibration model for the blood aliquot samples and chemical-extracted samples, the spectral ranges of 3100–2600 cm^−1^ and 1750–700 cm^−1^ were included, which involved more signatures in differentiating FM from other rheumatologic disorders. Blood aliquots model with five LVs for both FM and other rheumatologic disorder classes explained 91.54% of the variance and provided an excellent regression coefficient of cross-validation (Rcv) of 0.96. The calibration model for chemical-extracted blood samples with eight LVs explained 81.31% of the variance in both classes, providing an Rcv of 0.93. A cross-validated LMF (washed semi-permeable membrane filtration extraction) model was generated by seven LVs in two classes with a spectral range of 4000–2421 cm^−1^ and 1840–700 cm^−1^, which explained 83.17% of the variance with an excellent Rcv of 0.99. Similarly, the calibration model of LMF with glycerol with eight and seven LVs for FM and the other disorders, respectively, (3000–2380 cm^−1^ and 1881–1154 cm^−1^), explained 86.43 and 84.63% of the variance, respectively, with an Rcv of 0.99. OPLS-DA, with up to eight factors and one OSC removed, distinguished FM and other rheumatologic disorders with no misclassification for leave-one-out models.

The predictive accuracy of these calibration OPLS-DA models generated from the spectra with different sample preparation approaches was externally evaluated by the independently unseen 25% of spectral data. The accuracy illustrated the capability of the predictive models to differentiate the subjects with FM and the subjects with other rheumatologic disorders to the corresponding classes correctly. Sensitivity demonstrated the ability of the calibration models to determine the subjects with FM correctly, while specificity evaluated the performance of our model in determining the subjects with other rheumatologic disorders correctly [51]. External validation separated the spectral data from subjects with FM and subjects with other rheumatologic disorders, with excellent accuracy/sensitivity/specificity (Table 2) of 80.9%/86.7%/70.6%, 95.7%/93.3%/100%, 93.6%/93.3%/94.1%, and 83.0%/83.3%/82.3%, respectively, by the OPLS-DA models generated by samples prepared from blood aliquots, chemical protein-precipitated extraction, and unwashed and washed semi-permeable membrane extraction methods. Comparatively, models generated from protein-precipitated samples and LMF (washed semi-permeable membrane extraction) demonstrated a higher accuracy, sensitivity, and specificity than the models of blood aliquots and LMF with glycerol. 

**Figure 5:**
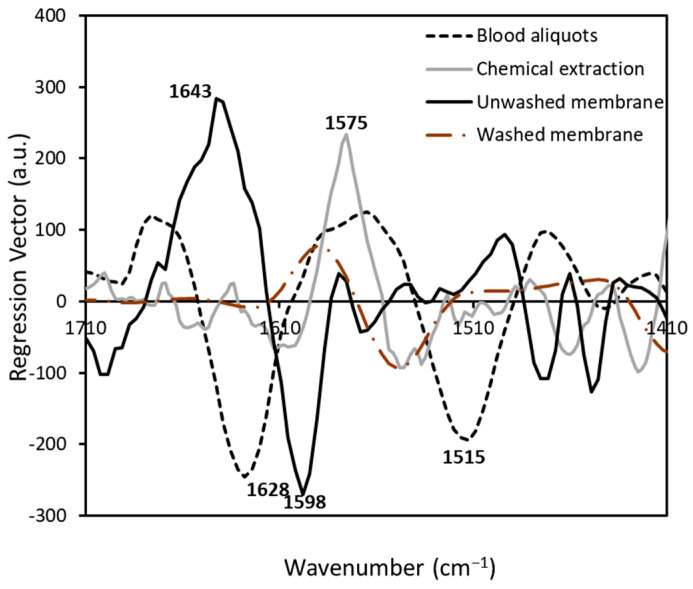
The regression vector plots of OPLS-DA regression models obtained from the spectral data of samples prepared by redissolving blood aliquots, chemical protein-precipitated extraction, washed membrane ultrafiltration extraction (LMF), and unwashed membrane ultrafiltration extraction (LMF with glycerol).

As significant as the predictive accuracy is the biological interpretation of the classification models. OPLS-DA calculated a regression coefficient for each variable, which represented the contribution of each variable to the discrimination of FM and other rheumatologic disorders (RA, OA, and SLE) [52]. Regression vectors from 1710–1410 cm^−1^ obtained from OPLS-DA models are shown in Figure 5. Positive peaks suggested positive correlations, while negative peaks indicated negative correlations, and zero represented no effect [39]. The regression vectors showed the discriminating region was dominated in 1710–1510 cm^−1^ by the bands centered at 1643, 1628, 1598, 1575 and 1515 cm^−1^, characteristic of C=O stretching vibrations in Amide I, the β-sheet structure, the N-H bend, the C–C aromatic ring, and the C=C stretch of aromatic compounds, respectively [5,53,54,55,56]. Furthermore, amide bands and aromatic rings in the profiles of the regression vector for all four models were consistently important.

Overall, multivariate analysis of IR spectra from different sample preparation approaches yielded robust models, reflecting that peptides in the blood fluid can be candidate vibrational biomarkers in the diagnosis of FM and other rheumatologic disorders (RA, OA, SLE). Furthermore, the importance of aromatic compounds was also highlighted by the chemometrics, which is in agreement with the finding reported by Hackshaw et al. [5], supporting that aromatic amino acids (i.e., tryptophan) can be candidate biomarker molecules. Our approach provided portable sensing capabilities to reduce the assay time and help streamline the diagnosis procedure, enabling real-time and field-based measurements at clinics and point-of-service.

## 4. Discussion

This study assessed the feasibility of a portable FT-IR spectrometer in the diagnosis of individuals with fibromyalgia as distinct from those with other rheumatic disorders, including RA, SLE, and OA. The different sample preparation approaches of human blood samples were evaluated and investigated in building predictive algorithms. The results of this study demonstrated unique IR spectral signatures that clustered subjects into the corresponding classes (FM and other rheumatic disorders (RA, SLE, and OA)) with good sensitivity and specificity. The regression vectors predominated by amide bands and aromatic ring structures, indicating peptides and aromatic amino acids in the blood can be candidate biomarkers. 

Different preparation approaches for serum samples have been evaluated in this study, and we found that the spectral profile of chemical-precipitated plasma and LMF prepared by washed membrane filtration was similar. A recent study reported by Gowda et al. has comprehensively compared the performance of protein precipitation by chemicals and ultrafiltration approaches using NMR. The ^1^H NMR of both proteins precipitated, and ultrafiltered serum detected all metabolites with comparable reproducibility. However, in ultrafiltered serum, nearly half of the quantified metabolites exhibited lower concentrations, especially tryptophan, benzoate, and 2-oxoisocaproate, compared to protein-precipitated serum [57]. In addition, Gekko et al. and Yadav et al. have found that with the addition of acetonitrile to an aqueous solvent, peptide-peptide hydrogen bonds could be enhanced, and the conformation of a protein (i.e., lysozyme) could change to a helix-rich form [58,59].

The success of developing OPLS-DA predictive algorithms demonstrated the capability of using non-targeted portable fingerprinting techniques to differentiate individuals with FM from those with RA, SLE, and OA. According to the studies reported by the American College of Rheumatology, the diagnosis accuracy and sensitivity of patients with fibromyalgia and other rheumatologic pains (but not fibromyalgia) were 84.9% and 88.4%, which used the traditional diagnostic approach by the pain analysis in up to 18 pain sites [60]. Comparatively, models generated from protein-precipitated samples and washed-membrane filtrated samples showed better predictive performance with high accuracy and sensitivity. The slightly lower/comparable accuracy and sensitivity of the models generated by blood aliquots and LMF with glycerol could be due to the large molecules that mask the significant fingerprinting information of the metabolites (biomarkers). 

Lechowicz et al. and Hackshaw et al. indicated that proline and tryptophan amino acids could be effective compounds in distinguishing RA from the control healthy group and FM from RA, SLE and OA groups, respectively, in agreement with our finding from multivariate data analysis that aromatic compounds/amino acids can be candidate biomarker molecules [5,61]. Interestingly, as discussed above, the ultrafiltered serum could have less tryptophan compared to protein-precipitated serum, and we also found the algorithm developed by ultrafiltered samples has a slightly lower sensitivity compared to the algorithm of protein-precipitated serum. 

Very limited studies have been performed by infrared spectroscopy to diagnose fibromyalgia previously. Recently, our group has investigated the metabolite profile of patients with FM (n = 50), RA (n = 29), OA (n = 19) and SLE (n = 23) using portable FT-IR spectrometer based on an ultrafiltration sample preparation method, where soft independent modeling by class analogy (SIMCA) was applied to discriminate different syndrome with 100% accuracy (20% samples for external validation) [5]. With the increasing sample number, OPLS-DA was selected in this study, which “combines the separation strengths of PLS-DA and SIMCA”, separating predictive from non-predictive (orthogonal) variation [62]. Similarly, Passos et al. have utilized benchtop FT-IR spectroscopy combined with GA-LDA (genetic-algorithm-based linear discriminant analysis), achieving an accuracy of 84.2% and sensitivity of 89.5% for differentiating blood plasma from fibromyalgia patients and control healthy subjects [60]. 

The clinical groups were generally similar in terms of BMI. The FM and SLE groups were similar in age, with RA and OA groups being generally older. FIQR scores, although not directly analogous to SIQR, do reflect the level of generalized anxiety and/or distress exhibited by individual subjects. As a result, FIQR (and SIQR) might represent surrogate measures of quality of life (QOL) for affected individuals. As would be expected, in FM subjects, FIQR scores were generally higher than the corresponding SIQR seen in the RA or SLE groups, reflecting more adversely impacted QOL. OA, not surprisingly, had the lowest scores. Similarly, BDI scores tended to be higher in FM subjects (2 fold) relative to scores in RA, SLE, and OA groups. Medications of the recruited patients have been recorded at the time of blood collection in this study. Based on the spectroscopy data, there was no obvious signal/influence from medications. However, the effect of medications was beyond the scope of this study. To determine the influence of medications on the results, further studies need a control population with medication free as well as similar clinical features and demographics in order to compare with the corresponding population with the medications. This would help to evaluate what effect, if any, medications might exert on spectral results. The analysis of Figure 2 reflects the individual FIQR values plotted against BDI scores, reflecting high dispersion within the FM group. The Pearson coefficient of 0.588 shows a moderate to strong association between the two variable scales, with evidence of a statistically significant association between the two continuous variables at the 0.01 level. The figure, however, reflects high dispersion reflective of the diverse phenotype that we see clinically in FM patients. Fibromyalgia patients have highly variable clinical presentations; some have a high degree of comorbid depression coupled with concurrent decreased overall quality of life (QOL), while others subjectively reporting high levels of depressive symptomatology may have their QOL less adversely impacted. Alternatively, we also encounter many individuals at the other ends of the spectrum and many with a mixed picture. Currently, we are conducting studies to metabolically evaluate the clinical domains of FM (quartiles of subjects based on depression levels, pain scores, and other validated measures) by vibrational spectroscopy. If metabolic differences between these domains can be ascertained by vibrational spectroscopy, then coupling this technology with complementary LC-MS/MS would provide valuable insights into targeted/personalized treatment approaches.

Many physicians may lack the training to diagnose FM accurately [5]. Patients with poorly explained symptoms are often inappropriately lumped into the FM category. For physicians, a diagnosis of FM often provides an explanation for difficult-to-understand symptoms [5]. Based on the results from the 2012 United States National Health Interview Survey, when patients received a diagnosis of FM, most of the patients did not satisfy published FM diagnosis criteria [63]. The latest diagnostic criteria still fail to provide an objective measure confirmatory of disease, which is actually what many FM patients are looking for. The identification of individuals with this clinical phenotype in different chronic pain cohorts can be a predictor of opioid non-responsiveness. The use of a rapid, reproducible biomarker can reassure patients that their symptoms have an objective marker and inform practitioners to direct therapy toward non-opioid regimens. Thus, this study has great significance in developing reproducible vibrational biomarkers for disease diagnosis and for identifying potential therapeutic targets. With the advances in the techniques discussed above, technologies for the diagnosis and treatment of FM and related rheumatic disorders might be advanced. 

## 5. Conclusions

In summary, this study showed that the in-clinic deployable fingerprinting FT-IR technique has the capability of discriminating individuals with fibromyalgia from those with other rheumatic disorders, including RA, SLE, and OA. Our results demonstrated the OPLS-DA algorithms developed by protein-precipitated and washed-membrane filtered samples have excellent sensitivity and specificity with no misclassification. Unique fingerprinting IR spectral signatures have been resolved by multivariate data analysis, and amide bands and aromatic ring structures dominated the regression vectors. Peptides and aromatic amino acids in the blood can be candidate biomarkers for syndromes such as FM. Ultimately, the development of robust predictive models based on portable IR spectra could allow for real-time and in-clinic diagnostics and potential therapeutic targets for potential revolutionary advances and huge cost savings.

## Figures and Tables

**Table 1:** Clinical characteristics of all subjects. Values expressed as mean +/−/sd; N = number of subjects, Age (range). FM: fibromyalgia, RA: rheumatoid arthritis, SLE: systemic lupus erythematosus, OA: osteoarthritis. BMI: body mass index. FIQR: fibromyalgia impact questionnaire revised. SIQR: symptom impact questionnaire revised. BDI: Beck depression index.

	Age R = 61 (18–79)	N (22/170-M/F)	BMI	FIQR	SIQR	BDI
FM	44.5+/−13.2	122	32.3+/−9.4	54.9+/−18.2		19.5+/−9.3
	R = 55 (18–73)	(M = 8, F = 114)				
RA	54.6+/−13.4	43	31.4+/−8.2		34.6+/−25.4	9.5+/−7.9
	R = 57 (20–77)	(M = 10, F = 33)				
SLE	43.9+/−15.2 R = 50	17	29.9+/−8.9		35.4+/−28.9	10.9+/−10.7
	(18-68)	(M = 1, F = 16)				
OA	63.5+/−8.0	10	35.8+/−9.9		27.4+/−18.9	7.3+/−6.3
	R = 27 (52–79)	(M = 3, F = 7)				

**Table 2:** Statistical performance results of OPLS-DA models obtained from the portable FT-IR spectral data of samples prepared by four different approaches (blood aliquots, chemical solvent precipitation extraction, and unwashed and washed semi-permeable membrane extractions).

Model Types	Accuracy (%)	Sensitivity (%)	Specificity (%)
Blood aliquots	80.9	86.7	70.6
Chemical precipitation	95.7	93.3	100
Washed membrane	93.6	93.3	94.1
Unwashed membrane	83.0	83.3	82.3

## Data Availability

The data presented in this study are available on request from the corresponding author.
